# Conceptualising Four Categories of Behaviours: Implications for Implementation Strategies to Achieve Behaviour Change

**DOI:** 10.3389/frhs.2021.795144

**Published:** 2022-01-11

**Authors:** Per Nilsen, Sebastian Potthoff, Sarah A. Birken

**Affiliations:** ^1^Department of Medical and Health Sciences, Linköping University, Linköping, Sweden; ^2^Department of Social Work, Education and Community Wellbeing, Northumbria University, Newcastle upon Tyne, United Kingdom; ^3^Department of Implementation Science, Wake Forest School of Medicine, Winston-Salem, NC, United States; ^4^Wake Forest Baptist Comprehensive Cancer Center, Winston-Salem, NC, United States

**Keywords:** behaviour change, implementation strategies, individual-level influences, collective-level influences, conscious cognitive processes, non-conscious processing

## Abstract

**Background:** Effectiveness of implementation strategies is influenced by the extent to which they are based on appropriate theories concerning the behaviours that the strategies intend to impact. Effectiveness may be limited simply because the strategies are based on theories that are limited in scope or are derived from partially inaccurate assumptions about the behaviours in question. It may therefore be important to combine insights from various theories to cover the range of influences on the behaviours that will be changed.

**Aim:** This article aims to explore concepts, theories and empirical findings from different disciplines to categorise four types of behaviours and discuss the implications for implementation strategies attempting to change these behaviours.

**Influences on behaviours:** Multilevel influences on behaviours are dichotomized into individual-level and collective-level influences, and behaviours that are guided by conscious cognitive processes are distinguished from those that rely on non-conscious processing. Combining the two dimensions (levels and cognitive modes) creates a 2 x 2 conceptual map consisting of four categories of behaviours. Explicitly conceptualising the levels and cognitive modes is crucial because different implementation strategies are required depending on the characteristics of the behaviours involved in the practise that needs to be changed.

**Conclusion:** The 2 x 2 conceptual map can be used to consider and reflect on the nature of the behaviours that need to be changed, thus providing guidance on the type of theory, model or framework that might be most relevant for understanding and facilitating behaviour change.

## Background

Implementation science seeks to understand and contribute to closing the gap between research evidence (“what we know”) and current practise in various settings (“what we do”) by identifying what hinders the uptake of evidence-based interventions, programmes and services, and addressing determinants of implementation using implementation strategies. Although research evidence for some strategies to achieve an evidence-based practise (EBP) is increasing, the evidence of the effectiveness of strategies to change behaviours in health care is generally weak (1, 2). A recurrent finding is that it is difficult to change an ingrained practise even if current evidence is superseded by new evidence and relevant implementation strategies are used (3, 4). Ultimately, behaviour change remains a fundamental challenge that is at the heart of implementation science.

The lack of convincing evidence of the effectiveness of most implementation strategies (5) has often been attributed to insufficient use of theoretical approaches in implementation science (6). Responding to the call for increased use of theories, a common approach in recent years has been to select “off-the-shelf” theories and determinant frameworks, of which there are plenty in the field. However, theories are assumptions about a phenomenon (7), so the utility of a given theory depends on the extent to which the assumptions underpinning a particular theory provide a plausible explanation. Thus, the effectiveness of implementation strategies may be limited simply because they are based on theories that are limited in scope or are derived from partially inaccurate assumptions about the behaviours that the strategies are intended to influence. For example, selecting a theory that explains behaviour in terms of individual's autonomy and independent beliefs, attitudes and motivation to perform certain behaviours may not be an optimal basis for an implementation strategy if current practise is determined more by individual's automatically enacted habits or a strong professional culture that restricts expression of individual differences. Therefore, it is important to combine insights from various theories to cover the range of influences on the behaviours that will be changed.

In this paper, we argue that inappropriate assumptions about current practise might lead to strategies being directed at individuals when in fact behaviours are strongly influenced by collective-level factors. Research is scant in implementation science concerning whether and to what extent individuals act truly independently of collective-level influences such as the work climate or professional culture of which they are a part (8, 9). Strategies may also be based on incorrect assumptions that current practise relies on conscious cognitive processing although the behaviours may be largely habitual. The extent to which the behaviours depend on conscious or non-conscious cognitive processes is not commonly addressed in implementation science (10–12); e.g., whether the use of a certain evidence-based intervention is characterised by careful deliberation or if the use is more automatically enacted.

The aim of this paper is to explore concepts, theories and empirical findings from different disciplines to categorise four types of behaviours and discuss the implications for strategies attempting to change these behaviours. The concepts, theories and findings were primarily derived from research seeking to understand and explain behaviours, encompassing fields such as psychology, nursing, sociology, behavioural economics, organisation research, political science and other social sciences. Based on this research, we dichotomize multilevel influences on behaviours into individual-level and collective-level influences, and distinguish between behaviours that are guided by conscious cognitive processes and those that rely on non-conscious processing.

Explicitly conceptualising the levels and cognitive modes is crucial because different implementation strategies are required depending on the characteristics of the behaviours involved in the practise that needs to be changed to enable the use of a new intervention, programme or service. Hence, for strategies to be successful, they must account for whether current behaviours are deliberate or if they are more automatically performed, and under what circumstances collective-level influences such as pressure from a social group override individual's beliefs or goals. Combining the two dimensions (levels and cognitive modes) creates a 2 x 2 map consisting of four categories of behaviours. The map is intended to function as a tool for making one's assumptions about a practise explicit; explicit assumptions may be tested in part with strategies that are designed to address the degree to which behaviours are influenced by individual- or collective-level factors and to which they are conscious or non-conscious.

## Individual- and Collective-Level Influences on Behaviours

Socioecological models are used in many research fields, including human development, sociology and public health, to understand the dynamic interrelations among various individual and collective influences on individual's behaviours. The collective level is often referred to as the context or environment, surrounding or setting in which behaviours occur (13). These influences are typically conceptualised as being external to individuals although they become internalised over time (14, 15). In clinical practise, influences on practitioner's behaviours may emerge from within the individuals (e.g., their motivation regarding the use of a specific intervention), the profession one belongs to (e.g., other physician's expectations concerning the use of this intervention), the organisation in which one works (e.g., leadership directives about the use of the intervention) and authorities in the society in which one lives (e.g., policies and financial incentives to support the use of the intervention) (16, 17).

The dynamic between individual- and collective-level influences can be understood with reference to the concept of strong and weak situations in which individuals find themselves. Situationism proposes that strong situations exert pressure on individuals to behave in a certain way, yielding similar behaviours across people as individual patterns of thoughts, feelings and behaviours are suppressed, whereas weak situations contain little pressure as to an appropriate behaviour, allowing individual characteristics to be expressed (18, 19). Clinical guidelines, standardised care pathways and other evidence-based recommendations to facilitate adherence to best practises can be considered approaches to convert weak situations into strong situations. In fact, the evidence-based movement is premised on the importance of creating strong situations to avoid decisions based on health care practitioner's “unsystematic clinical experience” [(20), p. 2420].

## Individual-Level Conscious and Non-conscious Cognitive Processing Influences on Behaviours

Individual-level cognitions, i.e., the mental processes involved in gaining knowledge and comprehension, that influence behaviours have been described in various fields of research in terms of two modes of cognitive processing: conscious and non-conscious processes (21). The conscious cognitive mode is usually viewed as a deliberative and rational cognitive process, involving an evaluation of a behaviour based on some combination of utility, risk, capabilities and social influences before forming and acting on an intention (22). This type of goal-directed behaviour is contrasted with behaviours that are performed without much conscious awareness or deliberation, i.e., the result of non-conscious cognitive processing (21).

In clinical practise, health care practitioners' behaviours likely to depend on non-conscious cognitive processing tend to be those that are relatively simple and/or have been performed frequently in the past (23). These are behaviours for which practitioners have developed cue-behaviour associations, whereby a specific context (e.g., medical instruments) acquires the potential to initiate behaviour with little awareness or conscious control (24). Frequently performed and/or less complex clinical behaviours largely driven by non-conscious processes may include conducting routine physical examinations, carrying out hand hygiene procedures or prescribing common medications (25). Behaviours guided by conscious cognitive processes can be expected to be more complex and may not have been performed repeatedly in the past (26).

The two modes of cognitive processing are the focus of dual process models, which have been used in several fields, including psychology, sociology and behavioural economics. These models posit that behaviours result from the interplay of two cognitive processes operating in parallel. Kahneman (27) labels the two processes System 1 and System 2. The automatic System 1 is instinctive, representing a largely non-conscious cognitive process that occurs in response to external and internal cues, and relies on previously learned, ingrained heuristics. System 2 is slower and more deliberative. Analogously, the Reflective-Impulsive Model applies the terms impulsive and reflective for the two modes. The impulsive system drives behaviour based on past experience, which is automatically activated in memory through external and internal cues. The reflective system involves utilisation of stored knowledge about a behaviour (e.g., beliefs) and available situational information to execute a deliberate behaviour (28).

Dual process models also specify a number of boundary conditions that moderate the relationship between conscious and/or non-conscious processes, including cognitive load, stress and physical or emotional exhaustion (29). For example, there is strong evidence suggesting that conscious processing decreases steadily over a normal working day, as cognitive resources become depleted (30, 31).

## Collective-Level Conscious and Non-conscious Cognitive Processing Influences on Behaviours

Much of the research focus on behaviour change has been on individual's cognitions as influences on behaviours, but the two modes of processing can also be considered at a collective level, e.g., the team, profession, department, organisation and society of which individuals are a part. Such collective-level influences to behave in a certain way may be more important than individual's cognitions regarding the behaviour(s) in question, causing a health care practitioner to use an evidence-based intervention, programme or service despite being sceptical of its merits.

The relevance of collective-level influences on individual's behaviours can be explained with regard to the utmost importance of belonging to social groups. Substantial evidence suggests that social relationships are critical to our mental and physical well-being across the lifespan. Social groups fulfil a sense of belonging, which is a psychological need for survival (32). We are fundamentally a social species and our nature is to recognise, interact and form relationships with other people (33). Therefore, meeting the needs of a group may be more salient to the group members than their own individual needs. Collectivism is the extent to which members of a group view the group's needs as superordinate to their own needs and the extent to which group members wish to maintain strong, harmonious relationships with other members of the group (34). This means that health care practitioners may be inclined to comply with the opinions of colleagues in a multi-professional team, department management directives or accept the norms, values and expectations inherent in their profession to gain or maintain social acceptance even if the resulting behaviours go against their own beliefs or goals (35, 36). In consequence, individual's behaviours that appear to be self-determined and independent may be collectively influenced.

Behaviour change theories in psychology, which are commonly used in implementation science, tend to put limited emphasis on collective-level influences on behaviours, one reason being that the focus is on predicting behaviour rather than exploring it in detail. Still, social influences are acknowledged in many behaviour change theories, e.g., the concept of subjective norms in the Theory of Planned Behaviour (37) or social outcome expectancies in Social-Cognitive Theory (38). Self-Determination Theory also accounts for external influences on behaviours, describing a continuum of different underlying motivation for behaviours, with externally regulated behaviours being performed because of external demand or possible reward (39). However, there is a great deal of research in social psychology and other fields, such as education, sociology and political science, that places greater emphasis on collective-level influences on individual's behaviours.

There are numerous concepts that refer to collective-level cognitive processes. Culture is the shared norms, values and assumptions that influence the behaviours among members of a social group (40), where the social group may be, for instance, a team, profession or organisation (41–43). Institutional logics are conceptualised as values, assumptions and beliefs that provide meaning to social group's daily activity, organise time and space, and reproduce their lives and experiences (44). Organisational routines are defined as a collective-level process that involves a repetitive, recognisable pattern of interdependent actions involving multiple actors (45). There are also many other collective-level concepts, with different levels of theoretical development, e.g., habits of mind (46), collective climates (47), group thinking (48) and shared mental models (49).

Importantly, collective-level influences on behaviours can be conscious, but when they become internalised over time they also provide meaning on which individuals rely during non-conscious cognitive processing, yielding unreflected, more or less automatically enacted behaviours (50). Thus, culture and institutional logics may partially consist of non-conscious norms, values, assumptions and beliefs, which can yield taken-for-granted behaviours in social groups (43, 44), Further, routines may be performed non-consciously because individuals do not devote conscious attention to the actions they execute (17, 51).

## Four Categories of Behaviours

**Figure 1:**
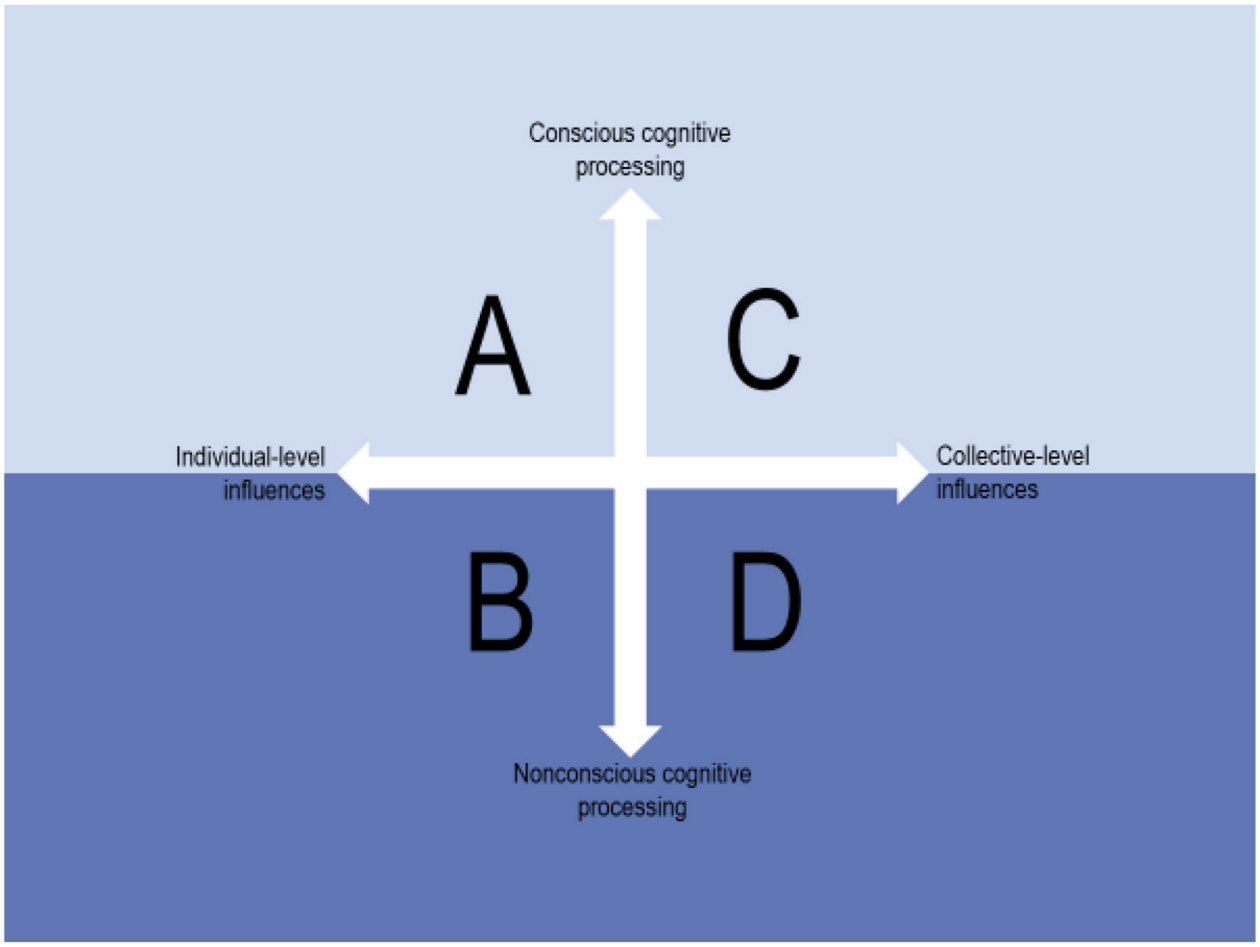
The 2 × 2 conceptual map of influences on behaviours.

Combining influences on practitioner's behaviours that emerge from the two levels (individual and collective) and rely on two modes of cognitive processing (conscious and non-conscious) creates a parsimonious 2 ×2 conceptual map (Figure 1). The four squares (i.e., different combinations of the two dimensions) represent four different conditions with regard to what influences practitioners' behaviours. Below the horizontal arrow, i.e., in the darker-coloured “water,” are behaviours that depend on non-conscious cognitive modes (squares B and D) and above the arrow, i.e., the lighter-coloured “sky” above the water, are those behaviours that are guided by conscious cognitive processing (squares A and C). The analogy is with figures of human consciousness (52) or organisational cultures (43) showing the non-conscious aspects underneath the water surface and aspects that represent conscious awareness above the water.

The 2 × 2 conceptual map represents a simplification of something quite complex. In practise, it may be difficult to determine the extent to which individual's behaviours are truly independent and based on individual-level influences, e.g., intrinsic motivation, or whether the behaviours in question are the result of internalised collective-level influences, e.g. collective routines that individuals have adopted. However, the 2 × 2 map points to the possibility of there being different types of influences on behaviours, therefore implying the need to tailor different implementation strategies to achieve the desired behaviour change.

It is unlikely that the four conditions exist on their own. Instead, the different types of influences are likely to work in parallel to influence practitioner's behaviours. There are also working conditions that may have an impact on the relative importance of the squares. For example, individual cognitions may exert a stronger influence on practitioners who work more independently compared with those who work in a close-knit team. Likewise, conscious processing may be more dominant in novel or unusual situations or where practitioners are new in their role. Non-conscious processing is likely more pronounced in experienced practitioners and/or in situations when cognitive resources are low, e.g., towards the end of a working day.

### Square A

Current practise in square A is characterised by behaviours that are under the control of individual conscious cognitive processing, with cognitions such as thoughts, attitudes, feelings, memory and motivation guiding their behaviours. Hence, individuals are independent decision-makers, with minimal or no collective-level influences on their behaviours. These behaviours are above the surface in Figure 1.

Empirical implementation science research suggests that many everyday clinical practise behaviours can be expected to fall into square A, particularly the behaviours of health care practitioners who are relatively new in their profession. As experience is built up, behaviours become less dependent on conscious cognitive processes. An example of a square A practise is diagnosing a clinical condition with which a health care practitioner is unfamiliar. A patient presents with a complaint and to diagnose the condition, the practitioner might need to undertake several activities: ask the patient to describe the symptoms; take the patient's health history; talk with the patient's caregivers; conduct a physical examination, laboratory tests, and/or imaging; and analyse the data collected (22). Each of these activities requires the practitioner to exercise conscious discretion.

### Square B

As with square A, individual's behaviours in a square B practise are influenced by individual factors, but the behaviours in square B rely on non-conscious cognitive processing, and thus are below the surface in Figure 1. In contrast to square A's example of diagnosing an unfamiliar clinical condition, a square B practise might include diagnosing a clinical condition with which a practitioner is highly familiar so that less cognitive effort is required to perform the task (25).

In general, ceasing or reducing the use of well-established, embedded interventions, programmes and services can present challenges as a result of the non-conscious cognitive processing involved in the behaviours, which have been performed frequently over time. This can be exemplified with the challenge of de-implementing prescribing inhaled corticosteroids for chronic obstructive pulmonary disease (COPD). Health care practitioners may automatically associate a COPD diagnosis with treatment involving an inhaled corticosteroid prescription (53). Another example is the use of computerised tomography (CT) for the evaluation of low-risk microscopic haematuria, a practise that exposes patients to high doses of radiation. Until May 2020, the American Urological Association recommended CT for the evaluation of all haematuria, but evidence suggests that revised recommendations have done little to decrease the use of CT (54).

### Square C

Current practise in square C is characterised by collective-level influences on behaviours, which are guided by conscious cognitive processes. These behaviours are above the surface in Figure 1. The members of social groups such as teams and professions are consciously aware of the collective forces that influence their behaviours in square C. Their awareness implies that behaviour change is easier to achieve than if practise resembles square D, where the collectively influenced behaviours rely on non-conscious cognitive processing.

Square C may be exemplified with regard to organisational routines in health care that are questioned or ignored, e.g., nurses deviating from the normal, embedded routine to sit with a patient to talk. They may be fully aware of the expected routine, but they want to do what they believe is appropriate at the time (55). Another example is the “culture of silence” that can develop among physicians in response to perceived expectations from colleagues to be highly skilled and faultless. This makes physicians reluctant to talk about errors they make even if they recognise that this culture is detrimental and that such discussions could yield learning for improved patient safety (36).

### Square D

Current practise in square D is characterised by internalised, collective-level influences on behaviours that rely on non-conscious cognitive processing, and thus are beneath the surface in Figure 1. Individual's behaviours in square D can be considered automatically enacted collective habits. This combination of non-conscious cognitive processing and collective-level influences on behaviours makes it difficult to change behaviours by using strategies that focus only on individuals and/or assume that the behaviours are guided by conscious cognitive processing.

Square D behaviours in the health care context may emerge from the same collective influences as those in square C, but with a difference in that the behaviours in square D rely on non-conscious cognitive modes. For example, organisational routines in nursing practise are often unquestioned and not reflected on, performed non-consciously to organise and coordinate activities (55). Other taken-for-granted and more or less automatically enacted behaviours may emerge due to the culture of the setting. For instance, the prescription of antibiotics may vary considerably due to hospital's different prescription cultures (56), and the use of surgical safety checklists can be entirely dependent on different safety cultures in operating rooms (57).

Square D behaviours in the health care context may also derive from unchallenged biases (preferences that interfere with the ability to be impartial) or broader stereotypes (over-generalised beliefs about a particular group of people), which might be activated involuntarily beyond individual's awareness (58). For example, physical pain among black people in the United States has often been found to result in under-treatment by white health care practitioners, something that has been attributed to an implicit racial bias (59). Similarly, the stereotype of “asexual old age” means that health care practitioners seldom address sexual health with older patients despite research showing that the elderly often have an active sex life (60).

## Discussion

Drawing on concepts, theories and empirical findings from several research fields and disciplines, the ambition of this article was to provide new perspectives on the challenges of changing behaviours to implement an intervention, programme or service. A 2 × 2 conceptual map was developed, consisting of four categories of behaviours. The map is *not* intended as a new theory, model or framework alongside the many already existing in implementation science and it cannot be positioned within the taxonomy described by Nilsen (6). Rather, the conceptual map can be applied to consider the nature of the behaviours that need to be changed to achieve an EBP, thus providing guidance on the type of theory, model or framework that might be most relevant for understanding and facilitating behaviour change.

The conceptual map can be seen as a tool for reflection on different types of influences on behaviours since such insights are important for determining which implementation strategies might be most relevant to change these behaviours. An understanding of current practise and the behaviours that need to be changed to implement evidence-based interventions, programmes or services can provide a more informed selection of strategies, thus increasing the likelihood of successful implementation. The map can be used to supplement established approaches to linking determinants with implementation strategies such as Implementation Mapping (61) and the Behaviour Change Wheel (62). Improved understanding of the determinants through the use of the conceptual map increases the likelihood of identifying relevant determinants and matching them with appropriate strategies.

To illustrate the applicability of the 2 x 2 conceptual map we use the example of implementing new evidence-based guidelines for acute stroke management within emergency departments (EDs) (63). Treating patients presenting to an ED with suspected acute stroke involves multiple health care practitioners (e.g., nurse, physician, endocrinologist, speech pathologist) working together in a standardised way to minimise harm to the patient. When new guideline recommendations are to be implemented one needs to consider each of the four categories of influences on behaviours.

In Square A (individual-level influences/conscious mode), to determine whether the health care practitioners have the necessary capabilities, opportunities and motivation to adhere to the guideline recommendations, one should consider collecting self-reported data directly with the practitioners [e.g., surveys (64) or interviews (65)] since they are aware of what they are doing and why. Strategies like education and training could be tailored depending on what might be most needed (66). In Square B (individual-level influence/non-conscious mode) one should consider observing health care practitioners' individual habits that may hinder adoption of the new guideline recommendations (12). For example, nurses may automatically manage patients' temperature with paracetamol at the wrong temperature threshold (63). Observational methods are useful because health care practitioners are unlikely to be able to articulate factors influencing their non-conscious behaviours. An audit and feedback strategy may be useful to bring non-conscious, undesirable behaviours into consciousness and substitute them with more appropriate behaviours in line with the guideline recommendations (67). In Square C (collective-level influences/conscious mode) one could consider using surveys with items referencing the group rather than the individual (e.g., “in the ED, we value…”) (68, 69) or conducting focus group interviews (70) to elicit collective perceptions of how health care practitioners collectively work together when trying to embed new guideline recommendations into their routine practise (71). Finally, in Square D (collective-level influences/non-conscious mode) one could observe ED operations to identify non-conscious collective routines that might exist among the health care practitioners that obstruct implementation of the new guideline recommendations (49). Observational methods could be used to explore collective influences on the ED staff's adoption of the guideline recommendations and make barriers and enablers explicit and therefore amenable if fed back to the health care practitioners and managers.

The 2 x 2 conceptual map complements T-CaST (“Theory Comparison and Selection Tool”) described by Birken et al. (72) to help researchers select appropriate theories, models or frameworks for their implementation project. T-CaST describes 25 criteria for assessment of the usability, testability, applicability and familiarity of theories, models and frameworks. However, it does not address whether or the extent to which a proposed theory, model or framework accounts for the characteristics of current practise and the underlying conditions for behaviour change. The 2 x 2 conceptual map could thus support the selection of theories, models and frameworks that, on their own or used together, provide the best understanding and facilitation of the behaviour change needed to achieve an EBP.

The first dimension of the 2 × 2 map distinguishes between individual-level influences (i.e., represented by squares A and B) and collective-level influences (i.e., represented by squares C and D) on behaviours. There is broad agreement in implementation science that behaviours are influenced by multilevel influences (often referred to as determinants) (6). The relevance of multilevel behavioural influences is evident in many theories and determinant frameworks applied in the field (13), e.g., Organisational Readiness to Change (73), Consolidated Framework for Implementation Research (74) and Exploration, Preparation, Implementation, Sustainment (75). The relative emphasis on individual- vs. collective-level determinants differs among these theoretical approaches. For example, the Theoretical Domains Framework's individual-level constructs are arguably its primary focus (12 constructs, including motivation, goals and memory), although it also includes two constructs at collective levels (environmental context and resources; social influences) (62). In contrast, Consolidated Framework for Implementation Research includes 16 constructs related to the inner and outer setting and just five constructs representing the individual level (74). Normalisation Process Theory is noteworthy for its focus on collective behaviours and the “collective action” that practitioners do when they implement a new intervention, programme or service (76). The deliberate combination of multiple theories, models and frameworks that address different levels may help to address the tendency for implementation strategies to target single levels of the socioecological framework, as Birken et al. (77) recommend in their systematic review of studies that combined Consolidated Framework for Implementation Research and Theoretical Domains Framework.

Collective-level concepts, such as culture, climate, leadership and resources, are part of many implementation theories and determinant frameworks (13). However, empirical studies in the field are usually premised on collecting data from individuals, typically on individual-level constructs such as attitudes, beliefs and intention concerning a specific behaviour, e.g., the use of an evidence-based guideline. The challenge of capturing collective-level influences is articulated in Jacobs et al.'s study (78) of implementation climate. Results were mixed with respect to the validity of using survey questionnaires to measure implementation climate based on aggregated individual responses (e.g., “I am rewarded for implementing the EBP”) vs. collective-referenced responses (e.g., “We are rewarded for implementing the EBP”). Fundamentally, aggregating responses may amount to reductionist fallacy, i.e., making inferences about group or other collective-level processes drawn from individual-level data (79). Avoiding this fallacy requires modes of data collection that do not focus on individuals. Ethnographic observational studies in clinical practise (80) and conversational analyses (81) may be routes to investigating collective-level influences on health care practitioner's behaviours, but such studies are rare in implementation science.

The second dimension of the 2 × 2 map distinguishes between conscious and non-conscious cognitive modes. At the individual level (i.e., represented by squares A and B), there is a substantial and accumulating body of empirical research that establishes that individual's cognitive processes can function outside conscious attentional focus (14). According to Greenwald and Krieger [(58), p. 945], the assumption that human behaviour is largely under conscious control (i.e., square A) has “taken a theoretical battering” in recent times. Interest in dual-processing approaches has increased significantly, with much attention focused on non-conscious processing. There is growing recognition that many behaviours are at least semi-automatic reactions to cues triggered by associations outside of conscious awareness and control (82). However, there is limited research concerning the extent to which this is the case in various practise settings or how it might have an impact on implementation of evidence-based interventions, programmes and services (25). Dual-processing models suggest that individuals do not always have conscious intentional control over cognitive processes and behaviours (83). As the saying goes, we are “creatures of habit” in many situations, pointing to the relevance of square B conditions for behaviour change.

The conscious/non-conscious cognitive processing distinction is usually not accounted for in implementation science theories or determinant frameworks. However, two notable exceptions are the Capability, Opportunity, Motivation–Behaviour (COM-B) theory and Theoretical Domains Framework, both of which distinguish between motivation to perform a behaviour that is guided by conscious and non-conscious cognitive processing (66). Theoretical Domains Framework thus acknowledges both dimensions (levels and cognitive modes) described in the 2 x 2 conceptual map, but it does not combine the two dimensions to describe four types of influences on behaviours. There are also a few empirical implementation science studies [e.g., (11, 25, 84)] that have sought to investigate the importance of the two cognitive modes for various behaviours contributing to an EBP.

The distinction between collective-level conscious and non-conscious processing, i.e., squares C and D, appears to be absent in studies in implementation science. Of course, collective-level influences such as a professional or organisational culture can be difficult to study because they partially depend on non-conscious cognitive processes, and are thus largely “invisible” and difficult to grasp. Schein (43) argues that members of a specific culture may need outside help to be able to make the shared tacit assumptions that make up the culture explicit. Similar challenges likely exist with regard to studying abstract collective-level concepts, such as institutional logics and routines, to explain their influence on individual's behaviours. Again, observational methods may be relevant to study collective-level influences and behaviours guided primarily by non-conscious cognitive processes. Such methods enable researchers to observe what people actually do (and not merely what they say they do) (71), which allows for exploration of group and other collective dynamics as well as studies of behaviours that are more unreflective and automatic.

In many ways, the implicit assumption in implementation science and practise is that current practise resembles square A. Hence, using information and various forms of training to influence individual's cognitive processing is likely the most prevalent type of strategy described in the literature (22). However, if the characteristics of current practise are more similar to the conditions of squares B, C and D, such strategies will be ineffective in influencing the practise. A fuller understanding of the conditions in squares C and D requires recognition of individuals as social creatures who build relationships of trust and cooperation within different social groups, e.g., the team, profession or organisation, and establish linkages that may strongly influence individual's behaviours. Understanding how individual's behaviours both shape and are shaped by social surroundings is a cornerstone of sociology, but studies of social relationships are also important in fields such as organisational theory, social psychology and evolutionary biology. To advance our understanding of the four squares proposed in this article, there is a need for more research that is multidisciplinary, combining different theories, to arrive at a fuller understanding of the challenges of behaviour change.

## Conclusion

Achieving EBP requires a clear understanding of the different types of influences on the behaviours that need to be changed. Drawing on concepts, theories and empirical findings from several research fields, this article demonstrates how influences on behaviours can be dichotomized into individual-level and collective-level influences, and describes the distinction between behaviours guided by conscious cognitive processes and those that rely on non-conscious processing. Combining the two dimensions, i.e., level and modes, creates a 2 × 2 conceptual map consisting of four categories of behaviours. The map can be used to analyse the characteristics of current practise and the nature of the behaviours that need to be changed. The map implies that knowledge about what influences the behaviours involved in a practise is important to guide the selection of relevant implementation strategies to change these behaviours. Very different implementation strategies might be required, depending on whether current behaviours are deliberate or if they are more automatically performed, and whether the behaviours are the result of internalised collective-level influences or if they are self-determined and independent, emerging from within the individuals.

## Data Availability Statement

The original contributions presented in the study are included in the article/supplementary materials, further inquiries can be directed to the corresponding authors.

## Author Contributions

PN conceptualised the study. All aspects were discussed with SP and SB. PN drafted the first version of the manuscript with assistance from SP and SB. All authors discussed further drafts, revised the manuscript, and approved the final manuscript.

## Conflict of Interest

The authors declare that the research was conducted in the absence of any commercial or financial relationships that could be construed as a potential conflict of interest.

## Publisher's Note

All claims expressed in this article are solely those of the authors and do not necessarily represent those of their affiliated organizations, or those of the publisher, the editors and the reviewers. Any product that may be evaluated in this article, or claim that may be made by its manufacturer, is not guaranteed or endorsed by the publisher.
